# Effect of Trabeculectomy on Mean and Centroid Surgically Induced Astigmatism

**DOI:** 10.3390/jcm11010240

**Published:** 2022-01-03

**Authors:** Wakako Ando, Kazutaka Kamiya, Masayuki Kasahara, Nobuyuki Shoji

**Affiliations:** 1 Department of Ophthalmology, School of Medicine, Kitasato University, Sagamihara 252-0373, Japan; wakako@kitasato-u.ac.jp (W.A.); m09078469240@hotmail.co.jp (M.K.); nshoji@kitasato-u.ac.jp (N.S.); 2 Visual Physiology, School of Allied Health Sciences, Kitasato University, Sagamihara 252-0373, Japan

**Keywords:** corneal astigmatism, surgical induced astigmatism, arithmetic mean, centroid, glaucoma, trabeculectomy

## Abstract

This study aimed to investigate the arithmetic mean of surgically induced astigmatism (M-SIA) and the centroid of surgically induced astigmatism (C-SIA) after standard trabeculectomy. We comprised 185 eyes of 143 consecutive patients (mean age ± standard deviation, 67.7 ± 11.6 years) who underwent trabeculectomy and completed at least a 3-month routine follow-up. In all cases, the scleral flap was made at the nasal-superior location. Corneal astigmatism was measured with an automated keratometer. We calculated the M-SIA and the C-SIA using vector analysis and applied the astigmatism double angle plot. The magnitude of corneal astigmatism increased significantly, from 1.17 ± 0.92 D preoperatively to 1.77 ± 1.05 D postoperatively (paired *t*-test, *p* < 0.001). The M-SIA was 1.12 ± 0.55 D, and the C-SIA was 0.73 D @64° ± 1.02 D in the right eye group, and the M-SIA was 1.08 ± 0.48 D and the C-SIA was 0.60 D @117° ± 1.03 D in the left eye group. The C-SIA showed an astigmatic shift toward the nasal-superior location of the scleral flap creation. Our results revealed that trabeculectomy induced the SIA in the direction of the scleral flap location and that the C-SIA was much lower than the M-SIA in eyes undergoing trabeculectomy.

## 1. Introduction

Glaucoma surgery is currently performed to lower the intraocular pressure (IOP) since high IOP is a major risk factor for glaucoma progression [1,2], and lowering IOP can slow down the progression of the disease [3,4]. Trabeculectomy has been widely acknowledged as the gold standard for glaucoma surgical interventions, possibly because of its excellent IOP-lowering effect over a long period. However, it is also known to be highly invasive due to the incisions in the conjunctiva, sclera, trabecular meshwork, and corneal limbus. The creation and the sutures of the scleral flap especially can result in a large amount of surgically induced astigmatism (SIA), resulting in significant deterioration in visual quality and subsequent patient satisfaction in post-trabeculectomy patients. Therefore, we should aim to reduce the IOP without the occurrence of postoperative complications, and to obtain good visual and refractive outcomes as much as possible, even in such glaucoma patients.

Currently, two analytic methods are available for SIA measurements in a clinical setting; the arithmetic mean of SIA (M-SIA) is calculated based on the mere magnitude of astigmatism, whereas the centroid of SIA (C-SIA) is calculated based on the magnitude, as well as the direction of astigmatism. Until now, several studies have been conducted on SIA outcomes after trabeculectomy, but most studies have merely focused on the M-SIA with a small cohort of such patients. Considering that the C-SIA was clinically helpful for grasping the overall trends of SIA, it may give us intrinsic insights on planning a preoperative strategy for reducing astigmatism in patients requiring trabeculectomy. Moreover, it is still difficult to accurately grasp SIA trends when using bilateral SIA data, since the scleral flap was made at different positions between the right and left eyes. The goal of the present study is to unilaterally evaluate both the M-SIA and the C-SIA in a large cohort of glaucoma patients undergoing standard trabeculectomy.

## 2. Patients and Methods

### 2.1. Study Population

This study was approved by the Research Ethics Committee of Kitasato University Hospital (B21-070) and registered with the University Hospital Medical Information Network Clinical Trial Registry (000045194). This retrospective review of the clinical charts was performed in accordance with the tenets of the Declaration of Helsinki. This study was carried out with explanations provided to the patients and a poster was displayed with additional information, including an opt out clause. Written informed consent for trabeculectomy was obtained from all patients after explaining the nature and possible consequences of the surgery. Our study comprised a total of one hundred eighty-five eyes of 143 consecutive patients (86 men and 57 women), who underwent standard trabeculectomy for the first time for glaucoma (primary open-angle glaucoma (POAG); 120 eyes and normal-tension glaucoma (NTG); 65 eyes), and who completed at least a 3-month follow-up. We excluded eyes with irregular corneal astigmatism, eyes with poor fixation at the time of preoperative or postoperative examination, or eyes requiring additional interventions, except for suture lyses by the argon laser, from the study. Fifty age- and gender-matched ophthalmologically healthy eyes were used as a control group.

### 2.2. Surgical Procedure

All surgeries were performed by two experienced surgeons (MK and NS) at Kitasato University Hospital. The scleral flap was created with a single half-thickness flap of approximately 3 × 3 mm^2^ (square) on the nasal-superior side through a fornix-based conjunctival incision in all cases. We unified the flap location in the 1 and 11 o’clock positions in the right and left eye groups, respectively. Afterward, 0.05% mitomycin C was applied for 3 min. A scleral window of approximately 2.0 × 0.5 mm^2^ (square) was created using a straight knife and small scissors. After peripheral iridectomy, the scleral flap was sutured with 4 10-0 nylon sutures. Continuous 10-0 nylon sutures were placed on the radial incision, interrupted sutures, and compression sutures were placed on the corneal limbus. If the achieved IOP exceeded the targeted IOP, laser suture lysis was performed as needed. Scleral flap suture lysis was performed by the argon laser based on the targeted and achieved IOP levels during the early postoperative period.

### 2.3. Assessment of Corneal Astigmatism and Surgically Induced Astigmatism

Preoperatively and 3 months postoperatively, corneal astigmatism was measured with an automated keratometer (TONOREFF-II, Nidek, Gamagori, Aichi, Japan). The average value from at least 3 reliable measurements was used for statistical analysis. Both the M-SIA and the C-SIA were determined by vector analysis of corneal astigmatism. Subjective and objective refractive SIA was calculated using the M-SIA, the C-SIA, and the paraxial approximation. The paraxial approximation is more precise and superior in principle, involving fewer approximations, and is not subject to systematic bias [5]. Double angle plots for the display of the individual SIA distributions were entered in the astigmatism double angle plot tool available on the American Society of Cataract and Refractive Surgery (ASCRS) website (https://ascrs.org/tools/astigmatism-double-angle-plot-tool (accessed on 31 October 2021)) [6].

### 2.4. Repeatability of Corneal Astigmatism Measurement

In order to confirm the repeatability of the measurements, corneal astigmatism measurements using the automated keratometer were made at the same time of the day on the two consecutive days in 40 pre-trabeculectomy eyes. The repeatability of the two measurements was evaluated using Bland–Altman plots, as described previously [7].

### 2.5. Statistical Analysis

The Shapiro–Wilk test firstly checked the normality of all data samples. Since we confirmed the normal distribution of the data, the paired *t*-test was used to compare the preoperative and postoperative data, and the Pearson correlation coefficient was used to assess the relationship of the two variables. The resultant values were expressed as mean ± standard deviation, and values of *p* < 0.05 were deemed statistically significant.

## 3. Results

**Figure 1:**
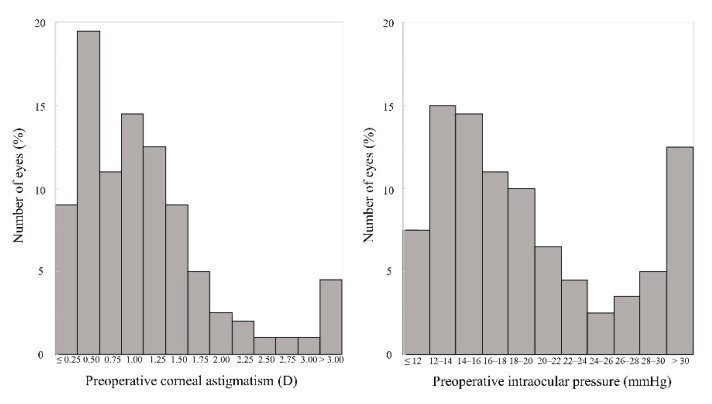
Graph showing the distributions of the preoperative corneal astigmatism and the intraocular pressure.

Figure 1 shows the distributions of the preoperative corneal astigmatism and the preoperative IOP. Table 1 shows the preoperative and the 3-month postoperative clinical outcomes of the study population. The IOP decreased significantly, from 21.4 ± 9.7 mmHg preoperatively, to 10.9 ± 4.6 mmHg postoperatively (Paired *t*-test, *p* < 0.001). Nine eyes (5%) showed a low IOP (<5 mmHg), but no eyes developed hypotonic maculopathy during the observation period. We found no significant differences in most parameters between the study and the control groups, except or the IOP (*p* < 0.001) (Table 1).

**Figure 2:**
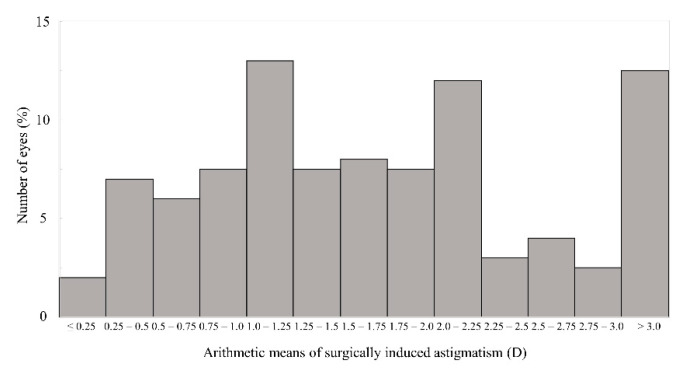
Graph showing the distributions of the arithmetic mean of surgically induced astigmatism (M-SIA).

**Figure 3:**
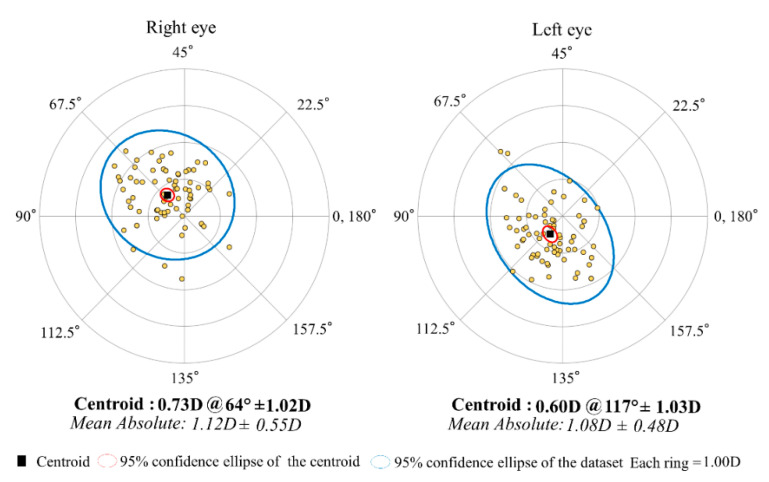
Graph showing the corneal SIA of trabeculectomy with double angle plots in the right and left groups.

**Figure 4:**
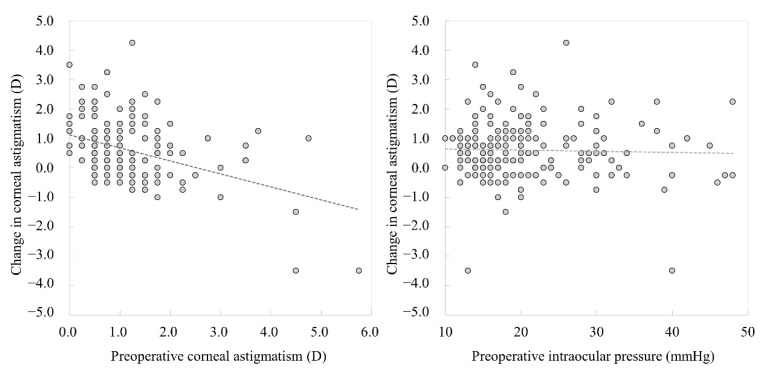
Graphs showing a significant correlation of the M-SIA with the preoperative corneal astigmatism (Pearson correlation coefficient *r* = −0.308, *p* < 0.001), but not with the preoperative intraocular pressure (*r* = −0.017, *p* = 0.823).

**Figure 5:**
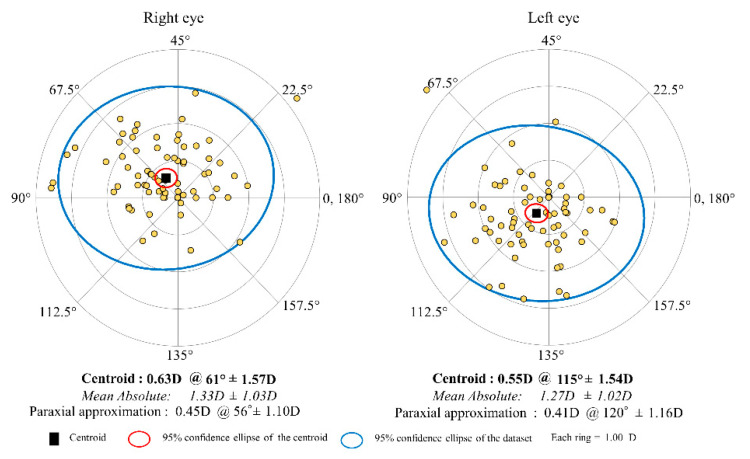
Graph showing the subjective refractive SIA of trabeculectomy with double angle plots in the right and left groups.

**Figure 6:**
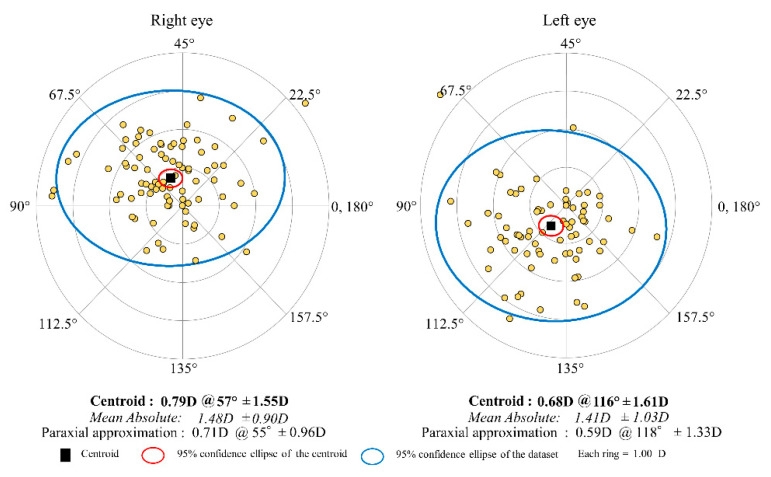
Graph showing the objective refractive SIA of trabeculectomy with double angle plots in the right and left groups.

Figure 2 shows the distributions of the M-SIA. Corneal astigmatism significantly increased, from 1.17 ± 0.92 D preoperatively, to 1.77 ± 1.05 D postoperatively (*p* < 0.001). In the right eye group, the M-SIA was 1.12 ± 0.55 D, and the C-SIA was 0.73 D @64° ± 1.02 D. In the left eye group, the M-SIA was 1.08 ± 0.48 D and the C-SIA was 0.60 D @117° ± 1.03 D (Figure 3). Although the double angle plots of individual SIA showed some dissimilarities in astigmatic magnitude and direction, the direction of the C-SIA exhibited a trend of corneal steepening to the nasal-superior location, not only in the right eye group but also in the left eye group. We found a significant correlation of the M-SIA with the preoperative corneal astigmatism (Pearson correlation coefficient *r* = −0.308, *p* < 0.001), but not with the preoperative IOP (*r* = −0.017, *p* = 0.823) (Figure 4). Figure 5 and Figure 6 show double angle plots of the changes in subjective and objective refractive astigmatism after trabeculectomy.

**Figure 7:**
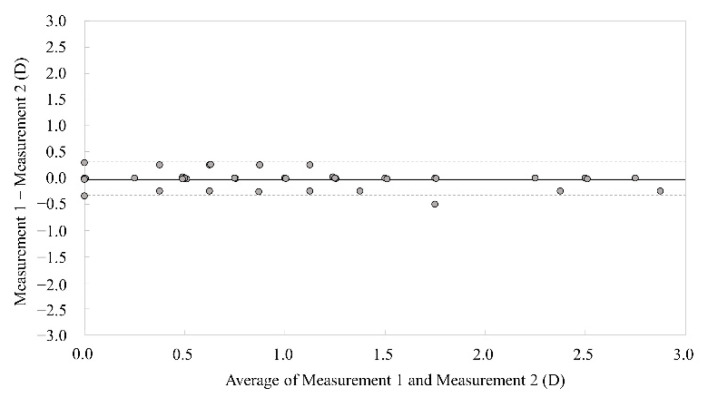
Bland–Altman plots showing the difference between two measurements divided by the mean of these astigmatic measurements in pre-trabeculectomy eyes. The solid lines represent mean differences between two consecutive measurements of corneal astigmatism, dotted lines are the upper and lower borders of the 95% LoA (mean difference ± 1.96 multiplied by standard deviation of the mean difference).

Bland–Altman plots indicate that the mean difference between the two measurements with this keratometer (±95% limits of agreement [LoA]) was −0.01 ± 0.14 D (−0.30 to 0.27 D) for corneal astigmatism (Figure 7).

## 4. Discussion

In the present study, our results showed that standard trabeculectomy significantly increased corneal astigmatism by approximately 0.6 D and that the surgery induced the M-SIA and the C-SIA by approximately 1.1 D and 0.7 D in the right eye group, and 1.1 D and 0.6 D in the left eye group, respectively. Our results also showed a significant correlation of the M-SIA with the preoperative corneal astigmatism, but not with the preoperative IOP, suggesting that preoperative corneal astigmatism might play a major role in the M-SIA after trabeculectomy, rather than the preoperative IOP. We assume that unilateral data analysis is more relevant and reasonable than bilateral analysis for the assessment of the actual SIA in daily practice. To the best of our knowledge, this is the first study to unilaterally demonstrate the magnitude and the direction of SIA in a large cohort of patients undergoing trabeculectomy. It has been reported that standard cataract surgery through a 3 mm corneal incision caused a M-SIA of approximately 0.5 D [8,9,10,11,12,13]. We previously revealed that the C-SIA of cataract surgery and phakic intraocular lens implantation was far smaller than that of the M-SIA [13,14]. In the current study, our findings demonstrate that the C-SIA was much smaller than the M-SIA in post-trabeculectomy patients, possibly because individual SIA showed variations in astigmatism in magnitude and direction in these patients. Both SIA findings of trabeculectomy were in good agreement with our previous SIA findings of cataract surgery and phakic intraocular lens implantation [13,14]. Therefore, we believe that the C-SIA is theoretically beneficial to understand the overall trend of SIA. Therefore, the M-SIA may overestimate the C-SIA and should be understood with caution when considering optimized astigmatic correction even in post-trabeculectomy patients. Our results confirmed that the axis orientation of the corneal SIA was almost identical to that of the refractive SIA, although the magnitude of corneal SIA was slightly different from that of the refractive SIA. It is indicated that SIA was mainly derived from the corneal components, rather than the ocular components, in eyes undergoing trabeculectomy.

Table 2 summarizes previous studies on SIA outcomes in eyes undergoing trabeculectomy. Our findings were in line with earlier studies in terms of the amplitude of SIA, but the direction of SIA was totally different among previous studies, such as with-the-rule (WTR) shift [15,16,17,18,19,20], against-the-rule (ATR) shift [21,22], and oblique astigmatic shift (OBL) [23,24]. We assume that this discrepancy might be attributed to the location of the scleral flap as well as to the small sample size in previous studies. Indeed, the flap location has not been mentioned in some studies, and the sample size was somewhat limited in most studies. Considering that the scleral flap location is generally different in the right and left eyes, we meticulously unified the flap location of the scleral flap as the nasal-superior site for all surgeons and conducted unilateral analyses of SIA with a large cohort of patients undergoing trabeculectomy. Consequently, we found an apparent trend towards an astigmatic shift in the direction of the scleral flap creation, not only in the right eye group (axis 64°) but also in the left eye group (axis 117°). Accordingly, we assume that the flap location might play a key role in the direction of SIA in post-trabeculectomy patients.

Although the exact mechanism of SIA remains unclear, several possible explanations for SIA after trabeculectomy have so far been advocated, including tissue removal under the scleral flap [15], the mechanical strength of 10-0 nylon sutures [16], delayed wound-healing responses caused by the use of mitomycin C [25], scleral contraction due to excessive cauterization [17,26], the presence of large filtering bleb and ptosis [18], and low IOP [27]. In addition, the aqueous fluid dynamic flow in the anterior chamber and the time-dependent wound-healing responses might play some role in the M-SIA and the C-SIA in the present study. Further research on the exact mechanism of SIA after this glaucoma surgery is necessary to clarify this point.

There are at least two limitations to this study. Firstly, this study was performed in a retrospective fashion. Secondly, we applied an automated keratometer to assess corneal astigmatism, since it is most widely used in daily practice. Accordingly, we did not evaluate the amplitude of posterior corneal astigmatism in this study. It has been demonstrated that the magnitude of posterior corneal astigmatism (approximately 0.30 to 0.35 D) is far smaller than that of anterior corneal astigmatism, and the axis orientation of posterior corneal astigmatism was constant (ATR astigmatism) in most eyes [28,29,30], and that anterior corneal aberrations measurements overestimated the total corneal aberration in most eyes [30]. A prospective randomized controlled study using corneal tomographers would be ideal to confirm our SIA findings. 

## 5. Conclusions

In summary, our findings showed that trabeculectomy significantly increased the SIA in the direction of the scleral flap location in both eye groups and that the C-SIA was much smaller than the M-SIA, even after trabeculectomy. We believe that this information will be simple, but clinically beneficial, not only for reducing the amplitude of astigmatism but also for the preoperative planning of astigmatic correction in eyes undergoing trabeculectomy in advanced glaucoma patients. 

## Figures and Tables

**Table 1:** Preoperative and postoperative demographics of the study group undergoing trabeculectomy and demographics of the control group.

Demographic			*p*-Value *	Control	*p*-Value **
Number (right eye/left eye)	185 (90/95)			150 (77/73)	
Age (years)	67.7 ± 11.6 (95% CI, 45.0 to 90.3)			67.1 ± 13.7 (95% CI, 40.3 to 93.9)	0.481
Gender (male: female)	86:57			83:67	0.852
	Preoperative Postoperative (3 months)				
BSCVA (logMAR)	0.22 ± 0.46 (95% CI, −0.68 to 1.12)	0.24 ± 0.35 (95% CI, −0.45 to 0.93)	0.670	0.07 ± 0.09 (95% CI, −0.1 to 0.24)	0.646
Manifest spherical equivalent (D)	−3.09 ± 3.63 (95% CI, −10.22 to 4.03)	−2.83 ± 4.85 (95% CI, −12.33 to 6.68)	0.749	−2.77 ± 4.02 (95% CI, −10.67 to 5.12)	0.655
Mean keratometric readings (D)	43.99 ± 1.60 (95% CI, 40.86 to 47.13)	44.12 ± 1.63 (95% CI, 40.92 to 47.33)	0.462	43.82 ± 1.33 (95% CI, 41.21 to 46.44)	0.938
Intraocular pressure (mmHg)	21.4 ± 9.7 (95% CI, 2.4 to 40.3)	10.9 ± 4.6 (95% CI, 1.9 to 19.8)	<0.001	13.9 ± 0.3 (95% CI, 6.7 to 21.1)	<0.001

D = diopter, CI = confident interval, BSCVA = best spectacle-corrected visual acuity, logMAR = logarithm of the minimal angle of resolution. * Preoperative vs. Postoperative, ** Preoperative vs. Control.

**Table 2:** Previous studies on surgically induced astigmatism in eyes undergoing trabeculectomy.

Author (Year)	Eyes	Period	Instrument	Scleral Flap			MMC	Change in Corneal Astigmatism	M-SIA	C-SIA	Astigmatic Shift
				Location	Size (mm)	Suture					
Hugkulstone et al. (1991)	10	7 weeks	autokeratometer	-	5 × 5	2 or 5	N.A.	-	-	-	WTR
Cunlifee et al. (1992)	16	10 months	autokeratometer	-	5 × 3	2	N.A.	-	-	-	WTR
Rose et al. (1992)	8	3 months	topography	superior	2 × 3	3	N.A.	1.5 to 2.5 D	-	-	WTR
Claridge et al. (1995)	29	1 month	topography	-	4 × 3	2	N.A.	1.08 D	-	-	WTR
Kook et al. (2000)	18	12 months	autokeratometer	-	4 × 3	5	Yes	0.65 D			ATR
Egrilmez et al. (2004)	11	6 months	autokeratometer topography	-	4 × 4	2	No	-	1.25 ± 1.08 D/1.24 ± 0.96 D	0.75 D @172°/0.75 D @174°	ATR
Delbeke et al. (2016)	47	6 months	autokeratometer	-	5 × 4	2	Yes	-	0.50 D	-	WTR
Tanito et al. (2017)	20	3 months	autokeratometer	nasal-superior	3–4 × 3–4	4	Yes	-	1.01 ± 2.27	-	OBL
Kim et al. (2018)	51	12 months	autokeratometer	temporal-superior (right eye)/ nasal-superior (left eye)	4 × 3	-	Yes	-	0.82 D	-	WTR
Konopinoska et al. (2021)	38	6 months	autokeratometer	nasal-superior (right eye)/temporal-superior (left eye)	4 × 4	4	No	approximately 1.0 D	1.13 ± 0.93 D (preoperative) 1.20 ± 0.74 D (postoperative)	0.16 D@141 ± 1.5 D (preoperative) 0.39 D@0.29 ± 1.38 D (postoperative)	OBL
Current	185	3 months	autokeratometer	nasal-superior	3 × 3	4	Yes	0.60 D	1.12 ± 0.55 D (right eye) 1.08 ± 0.48 D (left eye)	0.73 D @64° ± 1.02 D (right eye) 0.60 D @117° ± 1.03 D (left eye)	OBL

N.A. = not available, D = diopter, WTR = with-the-rule astigmatism, ATR = against-the-rule astigmatism, OBL = oblique astigmatism.

## Data Availability

The date presented in this study are available on request from the corresponding author.

## Abbreviations

Intraocular pressure, IOP; mean surgically induced astigmatism, M-SIA; centroid surgically induced astigmatism, C-SIA; diopter, D; standard deviation, SD; with-the rule astigmatism, WTR; against-the rule astigmatism, ATR; analysis of variance, ANOVA; limits of agreement, LoA.
